# The North American Practitioner

**Published:** 1891-07

**Authors:** 


THE NORTH AMERICAN PRACTITIONER. The Journal of the Post-Graduate Medical School, Chicago. Published by Charles Truax, Greene & Co., 75 and 77 Wabash Avenue. $1.00 per year. Comes among our exchanges,

From its pages we glean much that is of value, and its low price should commend it to all physicians. We have always contended that we cannot fully appreciate the results of Hahnemannian treatment without knowing what is being done by the most advanced of the old school. With such a journal as this one can see what is being done by teachers of practitioners.

